# Maternal recall of exclusive and any breastfeeding duration during the first 6 months- an examination of retrospective accuracy at 12 months within a large prospective breastfeeding survey in Germany

**DOI:** 10.1186/s13006-025-00808-3

**Published:** 2026-01-08

**Authors:** Mathilde Kersting, Erika Sievers, Nele Hockamp, Philipp Hülk, Thomas Lücke

**Affiliations:** 1 Research Department of Child Nutrition, University Hospital of Pediatrics and Adolescent Medicine, St. Josef-Hospital Ruhr-University Bochum, Alexandrinenstraße 5, 44791 Bochum, Germany; 2 Haale, Germany; 3 University Hospital of Pediatrics and Adolescent Medicine, St. Josef-Hospital Ruhr-University Bochum, Bochum, Germany

**Keywords:** Exclusive breastfeeding, Any breastfeeding, Recall, Breastfeeding promotion, Breastfeeding support, Monitoring, Accuracy, Scenarios

## Abstract

**Background:**

Effective promotion and support of breastfeeding requires reliable data on breastfeeding duration, particularly of exclusive breastfeeding (EBF). Maternal retrospective recall of breastfeeding duration is often used to assess breastfeeding rates and trends in a certain population, but recall accuracy is limited by potential recall bias or imprecision of memory. Objective of this study was to examine mothers’ ability to accurately recall their breastfeeding duration at 12 months postpartum after individual recall periods of 6, 8 or 10 months.

**Methods:**

This assessment has been embedded into the web-based nationwide prospective survey on breastfeeding and infant nutrition SuSe-II 2017–19 in Germany. A set of 2–3 simple recall questions on duration of EBF (as defined by World Health Organization) and any breastfeeding (ABF) has been compared to prospective maternal reports of breastfeeding at 2, 4 and 6 months as reference.

**Results:**

Data pairs of recalled duration and reference duration were provided by 572 mothers for EBF and 163 mothers for ABF. On average, duration of EBF and ABF differs statistically significantly between methods, but deviations are small (2–3 weeks), and tend to increase with longer recall period. For both, EBF and ABF, at a tolerance level of ±2 weeks, the recall overestimates breastfeeding duration in about 50% of recall-reference pairs, while underestimation is scarce (about 10%) and about 40% of data pairs are accurate. In scenarios with a deviation tolerance of 1, 2, or 4 weeks, around 20%, 40% or 70% of recalls would be classified as accurate.

**Conclusions:**

Asking about duration of EBF and ABF at 12 months postpartum with a simple set of questions in a large sample of mothers was found to be suitable for assessing ABF and EBF duration within the first 6 months. Specific scenarios of tolerance levels suggest a deviation between recall and reference breastfeeding duration of ±2 weeks as feasible compromise between high accuracy and high proportion of acceptable recalls.

**Trial registration:**

German Clinical Trials Register (DRKS) (ID: DRKS00014601).

**Supplementary Information:**

The online version contains supplementary material available at 10.1186/s13006-025-00808-3.

## Background

Breastfeeding and human milk are the normative reference standard for infant feeding [1]. Exclusive breastfeeding (EBF) is recommended for the first 6 months of life, followed by continued breastfeeding along with age-appropriate complementary feeding [1–4]. However, breastfeeding recommendations have not yet been achieved worldwide [5]. Therefore, targeted promotion and support of breastfeeding is necessary, and the effects of such interventions need to be monitored adequately [5, 6].

In Germany, breastfeeding rates are not satisfactory [7, 8]. Although the two nationwide prospective surveys on Breastfeeding and Infant Nutrition (SuSe) show an increase of breastfeeding rates in the last two decades, still only 57% of mothers breastfed exclusively at 4 months and 9% at 6 months in the latest survey SuSe II 2017–19 [9]. A systematic breastfeeding monitoring in Germany was suggested [8, 10]. Breastfeeding monitoring has to be based on reliable information on breastfeeding duration, especially EBF [5, 6]. Data on breastfeeding duration can be collected by (repeated) prospective assessments of the actual breastfeeding status starting at birth or (one-off) retrospective maternal recalls over longer memory periods [6, 11–13]. In general, recalls imply a lower burden for participants and researchers and are easier to administer, especially on a population basis or for Public Health purposes.

Validity and reproducibility of breastfeeding recalls has been examined for decades [11, 13, 14], with widely varying results up to the last years [11, 13–21]. Differences between studies may be due to the heterogeneity of study designs, recall periods, applied recall questions, definitions of breastfeeding status or reference comparators.

The objective of this study was to examine retrospectively mothers’ ability to accurately recall their breastfeeding duration, especially EBF, at 12 months postpartum compared to prospective maternal reports of breastfeeding duration in the first 6 months. This was made possible by embedding a simple set of recall questions on duration of EBF which conform to definition of World Health Organization (WHO) and any breastfeeding (ABF) into the prospective design of the SuSe-II-study 2017–19 in Germany, thus taking advantage of a large study sample and an online tool that corresponds to the communication habits of today’s generation of young parents.

## Methods

### Study design

The SuSe-II-study combines a cross-sectional assessment of breastfeeding promotion in maternity hospitals with a prospective assessment of breastfeeding and infant nutrition in mother-infant pairs recruited in the participating hospitals [22]. Written informed consent of hospitals and mothers was a prerequisite for participation. The mother-infant cohort of SuSe II 2017–19 is the basis of this analysis. Criteria for participation were a healthy, full-term newborn, no admission to a newborn intensive care unit, sufficient maternal knowledge of the German language, access to a telephone, availability of an e-mail address [9].

Participating mothers received web-based questionnaires 5 times during the first year postpartum: at 2 weeks and 2, 4, 6 and 12 months. The 2-weeks questionnaire asked about the infant’s current feeding and retrospectively about feeding in hospital and at discharge. In addition, sociodemographic data and other potential determinants of breastfeeding were assessed. The 4 follow-up questionnaires were almost identical, asking about breastfeeding status and any other foods or fluids consumed by the infant at that time. In addition, at 12 months, all mothers were asked to recall their breastfeeding duration.

### Assessment of breastfeeding duration

Once a regular follow-up questionnaire in the SuSe-II web system indicated that a mother had stopped EBF or ABF since the regular previous report, a special short questionnaire was sent out immediately, targeting mainly at her EBF or ABF duration (EBF-Q or ABF-Q, resp). Due to the short time interval between stopping breastfeeding (specified for EBF and ABF) and receiving the EBF-Q or ABF-Q, the reported breastfeeding duration in the EBF-Q and ABF-Q served as the reference for the recall at 12 months.

The questions for the reference and the recall breastfeeding duration are presented in Table 1. Potential scenarios of the timing of the different questionnaires and resulting recall periods are depicted as *Additional file *1.

**Table 1:** Applied questions regarding the duration of EBF and ABF in the SuSe-II-study

1a. EBF-Q	“How many weeks did you exclusively breastfeed, i.e. give no milk other than breastmilk, no (semi-)solid foods and no other liquid?
1b. ABF-Q	“How many weeks in total did you breastfeed?”
2. Recall questions added to the 12-month regular questionnaire	2.1. “Was your child ever breastfed?” → “Yes”/“No”/“I don’t know”/“No answer”
	2.2. “Up to which month of life did you breastfeed your child?” → “I don’t know”/“No answer”/“My child was xx month(s) old when I stopped breastfeeding”
	2.3. “For how long did you exclusively breastfeed your child, i.e. without additional fluids (water, tea, juice), milk (other than breastmilk) or (semi-)solid foods?” → “It was never exclusively breastfed”/“I don’t know”/“No answer”/ “It was exclusively breastfed until xx months of life”

EBF, exclusive breastfeeding; ABF, any breastfeeding; Q, questionnaire

At 12 months, all mothers received the regular 12-month questionnaire and, as part of this, the recall questions (RQ) on breastfeeding duration. Depending on the individual breastfeeding history in the first 6 months, the mother had to answer between 1 to 3 questions (Table 1, *Additional file *1). Due to the prospective basic study design, the individual recall period varied between 6, 8 or 10 months.

### Definition of breastfeeding categories

Breastfeeding was categorized according to WHO as EBF: no liquids or solids other than breastmilk/human milk (except medicine/vitamin/mineral drops or syrups); ABF: breastmilk and other fluids (water, juice), milk (formula) or solids (complementary food/Beikost) [6].

### Statistics

Comparing the recalled duration with the reference duration permitted the analysis of a mother’s ability to recall her breastfeeding duration accurately after being exposed to a time effect, that varied between 10 to 6 months. The different temporal effects divided the mothers in the analysis into different subgroups of recall periods of breastfeeding duration.

All mothers with pairs of data (EBF-Q + RQ, ABF-Q + RQ) were primarily included in the evaluation. The effects of the different recall periods are analyzed separately for EBF and ABF according to the possible EBF-Q and ABF-Q times (at 2, 4, 6 months postpartum). The breastfeeding duration in months in the RQ was multiplied by the conversion factor 4.35 to get the reported duration in weeks [12].

Sample characteristics are presented separately for mothers with EBF and ABF duration data.

For the mothers’ responses on the questionnaires (EBF/ABF-Q and RQ), central tendency and distribution measures were calculated. Via a Wilcoxon-signed-rank-test for dependent samples, it was tested separately for ABF and EBF, whether the median reference breastfeeding duration from the EBF/ABF-Q differed significantly from the median recalled breastfeeding duration from the RQ. The differences in the mothers’ answers were also described by central tendency and distribution measures separately for the different recall periods.

The Kruskal-Wallis-Test for independent samples was used to test (separately for ABF and EBF), if the recall ability (the difference in the reference breastfeeding duration and the recall breastfeeding duration) differs significantly between the different recall periods. If so, a post hoc analysis with pairwise comparisons was executed. The significance-level was adjusted by Bonferroni-correction for multiple tests.

The decision to use nonparametric tests for the grouped data was made after the Shapiro-Wilk-Test indicated, that the assumption of normally distributed data had to be rejected for the tested groups.

In further analyses, the recall ability was categorized into 3 quality levels depending on the tolerated deviation of the recalled breastfeeding duration from the reference (up to ±1 week, up to ±2 weeks, up to ±4 weeks). Results are visualized through bar plots. Further bar plots provide a comparison between percentages of underestimation, overestimation and proper recall of breastfeeding duration for the different recall periods. The maximum allowed deviation in the answers that count as proper recall was set at ±2 weeks.

Graphs of the cumulative duration of EBF and ABF provide further insights into group averages of recall ability.

## Results

### Study sample

In the basic SuSe-II study, 109 maternity hospitals (out of a total of 692 invited hospitals) participated. During the recruitment period of mothers (January to March 2018), 3810 deliveries were recorded at the hospitals out of which 2831 were eligible [9] (*Additional file *2). A total of 962 mother-infant pairs participated in the first survey 2 weeks postpartum and 869 mother-infant pairs in the last follow-up 12 months postpartum, corresponding to a prospective compliance of 90.3%. In addition to the regular follow-up questionnaires 2, 4, 6 and 12 months postpartum, EBF-Q’s were obtained from 764 mothers and ABF-Q’s from 517 mothers. This data analysis included 572 mothers (59.5%) who had changed their EBF status and 163 mothers (16.9%) who had changed their ABF status within the first 6 months postpartum and for whom pairs of reference and recall breastfeeding duration were available.

Regarding sociodemographic factors of the subsamples of mothers with data on EBF and ABF, about 80–90% of the mothers were 30 years or older and had been employed before maternity leave, about two-thirds had a high education level, and about half were primipara. Regarding breastfeeding and birth, 80–90% of the mothers were informed about breastfeeding before giving birth, about two-thirds had a vaginal delivery and intended to fully breastfeed for at least 4 months (Table 2).

**Table 2:** Characteristics of mothers with information on duration of exclusive or any breastfeeding

Characteristics	Exclusive breastfeeding		Any breastfeeding	
	n = 572		n = 163	
	n	(%)	n	(%)
**Age (years)**^**1**^				
18–29	113	19.8	48	29.6
30–34	257	45.1	70	43.2
≥35	200	35.1	44	27.2
**School education**^**1**^				
Higher secondary	401	70.2	94	58
Secondary	140	24.5	51	31.5
Basic + other	30	5.3	17	10.5
**Parity**				
Primipara	288	50.3	95	58.3
Multipara	284	49.7	68	41.7
**Partnership**				
Permanent	559	97.7	160	98.2
Single	13	2.3	3	1.8
**Delivery**				
Vaginal	410	71.7	102	62.6
Caesarean section	162	28.3	61	37.4
**Informed about BF before birth**				
Yes/was already informed	501	87.6	138	84.7
No/other	71	12.4	25	15.3
**BF duration intention**^**2**^				
4 months or more	397	69.4	94	57.7
As long as possible	114	19.9	29	17.8
No idea/insecure	61	10.7	40	24.5
**Diet**				
Omnivore	543	94.9	161	98.8
Vegetarian/vegan	29	5.1	2	1.2
**Employed before maternity leave**				
Yes n	479	83.7	131	80.4
No	93	16.3	32	19.6
**Partner’s attitude towards BF**				
Positive	524	91.6	139	85.3
Other	48	8.4	24	14.7
**Infant’s sex**				
Male	288	50.3	85	52.1
Female	284	49.7	78	47.9
**Birth weight category (g)**				
≤3500	308	53.8	87	53.4
> 3500	264	46.2	76	46.6

BF, breastfeeding; ^1^ missing answers: age *n* = 3, school education *n* = 2; ^2^ “full” breastfeeding (question in the basic SuSe questionnaire: “Before giving birth, how long did you plan to fully breastfeed (i.e., without supplementing)?”

### Breastfeeding duration

Among mothers who had stopped EBF or ABF within the first 6 months of life, median duration of EBF was 20.0 (P25:13.0, P75: 23.0) weeks in the reference and 21.8 (P25:17.4, P75:26.1) weeks in the recall (*p* = 0.000), and median duration of ABF was 10.0 (P25: 4.0, P75:16.0) weeks in the reference and 13.1 (P25:4.4, P75:17,4) weeks in the recall (*p* = 0.000).

Analysing the three time periods separately confirms higher recalled duration than reference duration (*p* = 0.001) for EBF. In post hoc analyses with pairwise comparisons significant difference can only be confirmed for the 10 months vs. 6 months recall period (*p* = 0.003) (Table 3). The post hoc test result remains significant after the Bonferroni-correction. For ABF, no significant differences of breastfeeding duration between reference and recall specified for the different time periods are observed.

**Table 3:** Differences in breastfeeding duration between reference (EBF-Q/ABF-Q) and recall (RQ) assessment, categorized by the length of the recall period

	n	Median	P25; P75	Mean	SD	p-value
**Differences of answers (in weeks)** **(RQ – EBF-Q)**						
10 months	122	3.40	0.00; 12.40	6.21	8.75	
8 months	94	3.40	0.40; 5.93	3.85	6.33	
6 months	356	2.10	−0.25; 4.45	2.30	5.33	
Total	572	2.10	0.00; 5.70	3.39	6.55	0.001*
**Differences of answers in weeks** **(RQ – ABF-Q)**						
10 months	49	2.35	0.00; 3.35	2.56	3.40	
8 months	56	2.05	0.14; 4.05	2.13	3.24	
6 months	58	1.40	−0.25; 3.84	1.56	4.79	
Total	163	2.05	0.00; 3.70	2.01	3.90	0.584

n = number; Md, median; SD, standard deviation; P, percentile; RQ, reference questionnaire; EBF, exclusive breastfeeding; ABF, any breastfeeding; *Kruskal-Wallis-Test; 10 vs 8: *p* = 1.00, 8 vs. 6: *p* = 0.062, 10 vs. 6: *p* = 0.003

In both breastfeeding categories, differences between reference and recall increase with longer recall periods, especially for EBF.

**Fig. 1:**
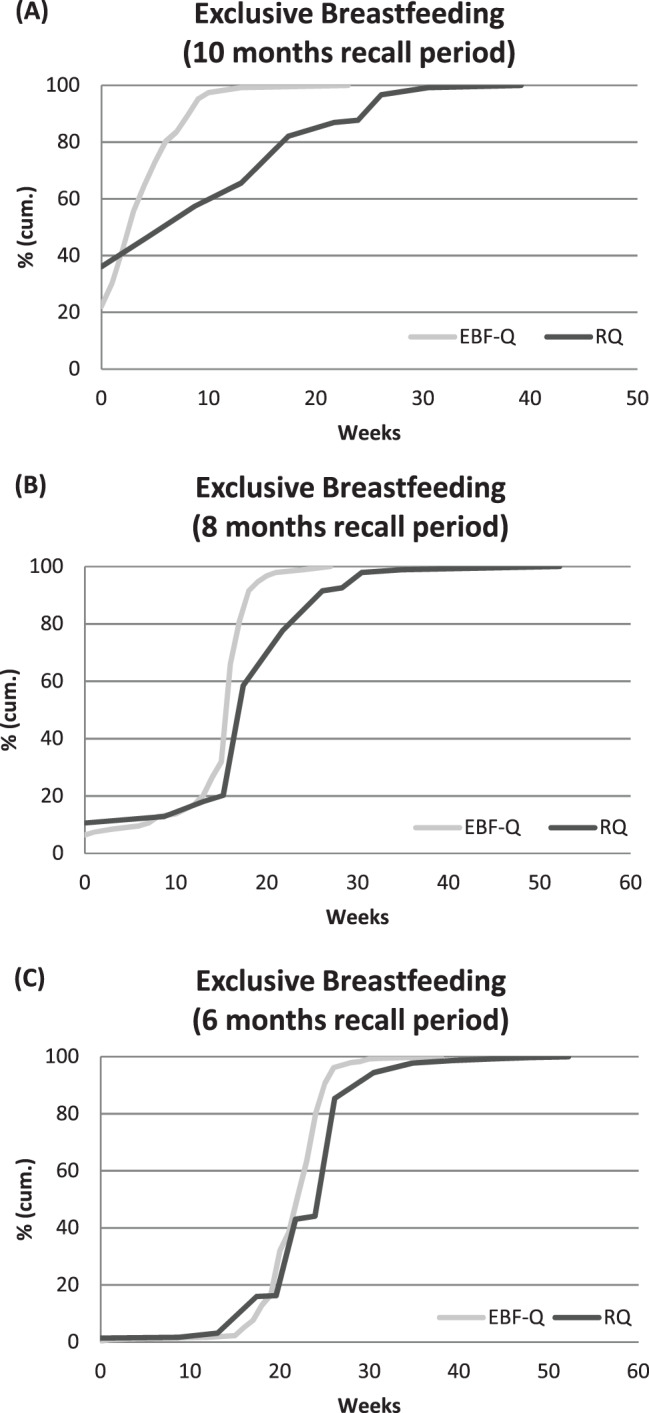
Comparison of the exclusive breastfeeding (EBF) duration (in weeks) given in the reference (EBF-Q) and the recall (RQ), specified for the recall periods 10, 8 and 6 months. Legend: cumulative percentages of exclusive breastfeeding (EBF) duration (weeks) given by mothers in the reference (EBF-Q) and in the recall (RQ), **A**: recall period 10 months (n: 122 mothers), **B**: recall period 8 months (n: 94 mothers), **C**: recall period 6 months (n: 356 mothers)

Maternal answers on breastfeeding duration are displayed by cumulative proportions of answers for EBF (Fig. 1). As an example, for the recall period of 10 months, the lower curve of the recalled duration (RQ) than the reference duration (EBF-Q) indicates a higher proportion of higher EBF durations in the recalls (Fig. 1A). With decreasing recall period (Figs. 1B, C) the proportions of durations given by mothers match better. Similar tendencies are found for ABF duration (data not shown).

### Scenarios of accuracy

**Fig. 2:**
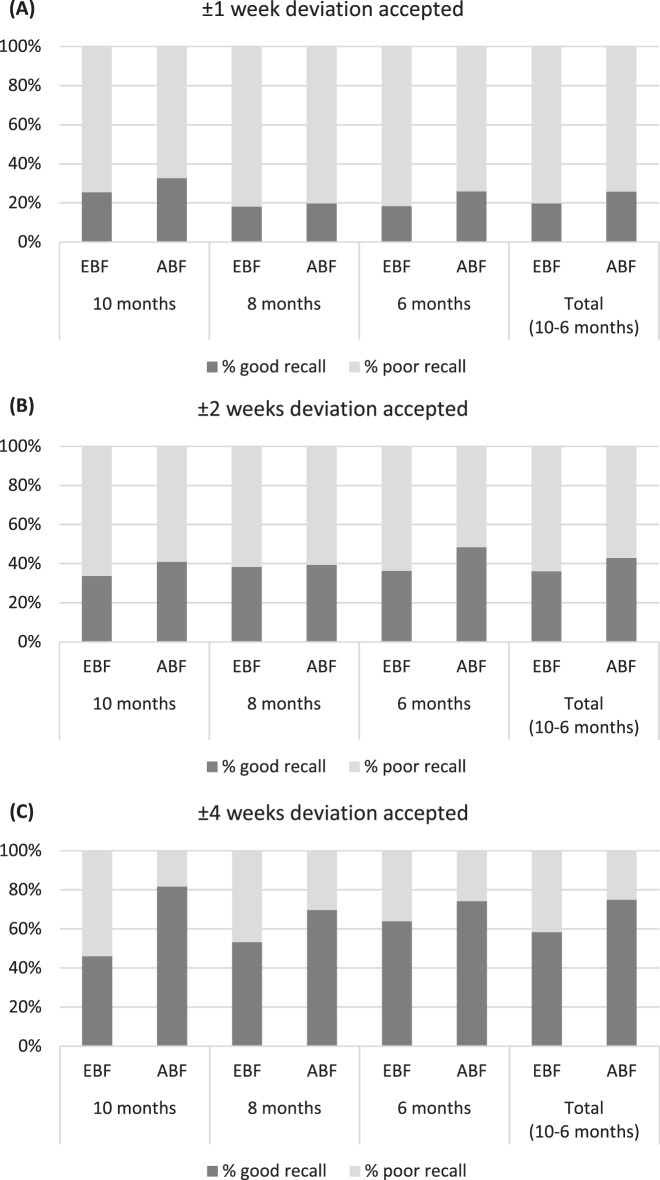
Percentage of ‘good’ and ‘poor’ recalls of exclusive (EBF) and any breastfeeding (ABF)- duration, at different levels of tolerated deviation from the reference. Legend: good recall: accuracy within the respective tolerated deviation; poor recall: poor recall: deviation larger than the respective tolerated deviation. **A**: tolerated deviation ±1 week, **B**: tolerated deviation ±2 weeks, **C**: tolerated deviation ±4 weeks. Study samples: EBF n: 572 mothers, ABF n: 163 mothers

Results of scenarios of accuracy at three levels of *tolerated deviation* from the reference are shown in Fig. 2 At a very strict tolerance level (±1 week deviation), about 20% of recalls would be rated as ‘good’, at a level of ± 2 weeks, the proportion of good recalls raises to about 40%; a generous tolerance of ± 4 weeks results in up to 70% ‘good’ recalls. Overall, these relations are largely independent of the breastfeeding category and the recall period.

**Fig. 3:**
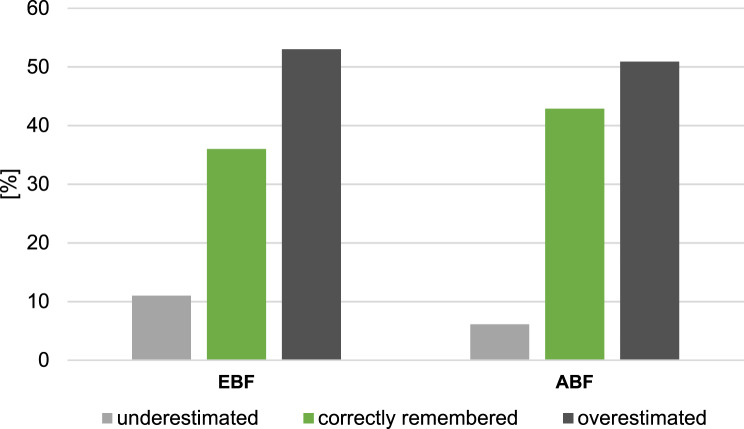
Direction of deviation (underestimation/overestimation) of the recalled duration from the reference for exclusive breastfeeding (EBF) and any breastfeeding (ABF). Legend: percentage of participants; summarized for the recall periods 10, 8 and 6 months; ±2 weeks = correctly remembered, overestimation/underestimation = higher/lower duration in recall than in reference study samples: EBF n: 572 mothers, ABF n: 163 mothers

An overview of the *direction of deviation* of the recall from the reference (at a tolerance level of ±2 weeks), demonstrates that for both, EBF and ABF, the recall overestimates breastfeeding duration in about 50% of recall-reference pairs, while underestimation is scarce (about 10%) and about 40% of data pairs are accurate. (Fig. 3).

## Discussion

### Overview

This evaluation of a set of simple retrospective questions on duration of EBF and ABF applied online 12 months postpartum in comparison with prospectively collected data in the first 6 months provides the following overarching findings:

- Quantitatively, breastfeeding duration differs statistically between the methods, but effectively differences are small compared to the recommended EBF duration; the accuracy of the recall tends to decrease with increasing duration of the recall period from 6 to 10 months.
- Qualitatively, data consistently confirms an overestimation of breastfeeding duration by recall.
- The tolerance scenarios as a specific feature of this evaluation clearly demonstrate how the tolerated level of deviation of the recall from the reference impacts on the rating of recall accuracy, thus helping users of the recall questions to interpret their results.
- Since recalling of EBF conform with WHO proved to be at least as accurate as recalling of ABF, the recall questions could be used especially for monitoring effects of interventions to improve EBF.

### Quantitative and qualitative findings

For surveying breastfeeding duration in larger population groups, retrospective recalls are an attractive, cost-efficient alternative to prospective studies. Potential limitations, e.g. recall bias, especially a tendency to answer conform to social desirability and the imprecision of memory [18] may impair their application [18, 23]. Specifically, the accuracy of recall depends on the ability of the mother to remember the week or month when EFB was stopped [6, 12]. Our overarching result of the recall as suitable for asking mothers retrospectively about the duration of breastfeeding is in line with diverse other studies even over longer recall periods [11, 13, 14, 16, 18] but differences between studies in various aspects need to be considered.

Quantitative differences of breastfeeding duration (weeks, months) between reference and recall assessment of this study are hardly to compare with other studies in the first years of life e.g. due to differences in study design, sample characteristics, recall periods [13, 15–17, 19, 20]. This heterogeneity exists in older studies (1966–2003) [11] as well as in more recent literature, exemplified by 3 studies: in the USA, Gillespie [24] asked a subsample of mothers (*n* = 184) of a larger study on breastfeeding habits, about the time they stopped ABF (month, day, year) prospectively in the first 3 months via weekly telephone calls (‘reference’) and retrospectively with a postal questionnaire at 6 months, 1 year, and 3.5 years. ABF duration (median) assessed prospectively was 2.8 months, however after a recall period of 3 months 3.2 months, and after 9 months 3.9 months. I.e. differences between reference and recall were greater compared to our study.

In Denmark, Bruun et al. [19] in a birth cohort (*n* = 639) compared weekly SMS questions (‘reference’) on breastfeeding duration with self-administered questionnaires at 3 and 18 months. EBF duration (mean) assessed at 3 months was almost the same with prospective weekly SMS (12.0 weeks) and with retrospective questionnaires (12.3 weeks) but much higher (19.0 weeks) with questionnaire at 18 months, i.e. after a recall period of about 15 months. ABF duration was the same in reference (SMS) and questionnaires at 18 months. In our sample, median difference between reference and recall was 3.40 weeks for EBF and 2.56 weeks for ABF at the longest recall period of 10 months.

In a population-based birth cohort (*n* = 3700) in Brazil, Schneider et al. [16] as part of a detailed in-person dietary assessment assessed EBF prevalence at 3 months (reference) and at 12 months, resulting in 27,8% EBF in reference (maternal report) and almost twice as high (49,0%) in recall at 12 months.

In the qualitative evaluation of the direction of difference, this study is well in line with most other studies finding an overestimation of breastfeeding duration in retrospective assessment, but degrees of overestimation differ between individual studies [14–17, 19, 20, 24]. The extent of overestimation increases with longer recall periods also in studies within the first years of life as in this study [13, 19, 24].

### Study design

In exploring the suitability of maternal recalls to assess breastfeeding duration, studies focused directly on this topic [14, 15, 17] or breastfeeding duration was part of broader investigation on breastfeeding or infant feeding [16, 18, 19, 24]. This exploration was embedded in the prospective design of the SuSe study on breastfeeding prevalences and associated factors during the first 12 months of the infant [9].

The basic SuSe study design favors realistic testing of potential questions in retrospective breastfeeding surveys: (1) the longitudinal approach with close-meshed questionnaires on breastfeeding duration immediately after a change in breastfeeding status within the first 6 months after birth enabled a robust reference for the recall after 12 months; (2) the timing of the reference data, in particular at 4 and 6 months after birth, reflects the introduction period of complementary feeding recommended by European authorities [25, 26], i.e. a change in EBF status; (3) the high longitudinal adherence of mothers up to the time of recall reduces the risk of bias due to loss of participants in long-term studies [27].

### Study samples

Results of exploring breastfeeding recall accuracy could be related to characteristics of the study sample.

With a nationwide sample of 735 mothers who provided the necessary data on pairs of methods, by far most on EFB, this study is one of the largest studies in this field of breastfeeding research carried out worldwide [16, 19, 24] and one of the few such studies in Europe [19, 21]. Sample sizes for BF vary between several thousand for EBF (e.g. 3700) [16] in Brazil, to around 600–700 in Denmark and USA [14, 19] and 100–300 in the USA, South Africa, Sri Lanka [15, 17, 20, 24].

Studies that included investigation of maternal characteristics potentially associated with the ability to remember breastfeeding duration over varying periods of time suggest that factors that are generally favorable for breastfeeding, such as a higher socioeconomic status, a healthy lifestyle, or prenatal breastfeeding information, may also favor breastfeeding recall ability [14–16, 18]. The extent to which such indications can be transferred to different sociocultural structures would need to be specifically investigated.

### Recall of EBF as a challenge

For the assessment of breastfeeding, the use of standardized definitions, e.g. that of WHO, enables meaningful interventions and measurement of their effects also in comparison with other studies [12]. Recall evaluations have tended to investigate ABF, which appears easier to define, ask about and remember than EBF [13, 18, 24]. For recall of ABF duration in this study, 2 simple questions were applied.

EBF is at the center of promotion and epidemiology of breastfeeding [5]. One key issue of assessing EBF is, whether mothers define the breastfeeding category themselves, or researchers categorize EBF post hoc by explicit exclusion of other liquids and food as in this and other studies [16, 17, 20]. In this study confined to EBF in the first six months, recall of EBF required that a mother had to be sure that her child had not yet been given ‘additional fluids (water, tea, juice), milk (other than breastmilk) or (semi-)solid foods’. In studies where EBF duration in the second half of the first year should also be recalled (when diverse complementary foods become common), greater efforts may be needed [16, 17, 20].

For monitoring the effects of interventions to increase EBF success of mothers it is advantageous that short term trends in breastfeeding can be identified, and effects of interventions can be monitored continuously. In this study sample, about 20% of mothers had even stopped breastfeeding completely within the first 6 months and about 40% had stopped EBF before 4 months postpartum. These mothers might have profited from effective interventions.

### Tolerance levels of accuracy

The scenarios with different tolerance levels of deviation of the recall from the reference are specifically featured in this analysis. The strong effects of the tolerance level on the probability of rating recalls as “accurate” could provide guidance for users of the recall. This applies e.g. for public health purposes of monitoring the effects of interventions to increase breastfeeding rates. As many barriers to breastfeeding exist at a societal level, breastfeeding promotion has been advocated as a public health issue [28].

The scenarios can also give an orientation about the reliability of individual breastfeeding anamnesis and counseling by medical or healthcare professionals.

A certain degree of overestimation of recalled breastfeeding duration should always be taken into account as this and other studies consistently show [14–17, 19, 20, 24]. The generally increasing misestimation of individual breastfeeding duration with increasing distance of the recall from the actual breastfeeding termination may at least partly be explained by increasing demands on memory. Another reason could be that the generally recommended or individually targeted goals of the duration of breastfeeding have not been achieved, and that disappointment is glossed over in retrospect [23].

### Limitations

Besides the strengths of this study, e.g. the large sample size, especially of mothers with data on EBF duration, the robust reference data from the prospective assessments in the early breastfeeding period and the application of WHO definitions for the breastfeeding indicators, limitations also need to be mentioned.

The maternal ability to remember their breastfeeding duration may have been favored by the fact that the mothers in this study had to repeatedly deal with breastfeeding reporting in the first 6 months as part of the regular SuSe surveys and this may have raised their awareness of breastfeeding even until 12 months after birth.

A limitation of this study is that mothers stated the duration of breastfeeding in weeks in the reference and in months in the recall. The problem of variations in breastfeeding timings in recommendations and practice assessments is well recognized in breastfeeding research. Recalculation procedures of time periods are not yet standardized [12]. By asking the duration of breastfeeding in the recall in months and posthoc recalculation in weeks (1 month = 4.35 weeks), we tend to underestimate the duration of breastfeeding compared to the specification in weeks in the reference. It is estimated that this underestimation would be less than two weeks for about half of the data pairs. The scenarios of different tolerance levels for deviations from the reference may give an orientation on the extent of potential misjudgements in breastfeeding recall.

In the basic SuSe-II-Study, mothers with a high education level were overrepresented [9]. The extent to which this may limit the transferability of the results would have to be investigated specifically.

## Conclusion

The simple set of recall questions applied online 12 months postpartum proved to be suitable to assess the duration of EBF and ABF within the important first 6 months. An overall tendency to overestimate breastfeeding duration by mothers in retrospect should always be taken into account even in short recall periods of 6–10 months. The strong effects of different tolerance levels of accuracy on the proportion of acceptable recalls shown here can help in the planning of interventions to improve breastfeeding rates and to monitor effects in short intervals. In principle, the results of this study could be transferable to countries with similar structures of maternal and child care as in Germany.

## Electronic supplementary material

Below is the link to the electronic supplementary material.

Supplementary Material 1

Supplementary Material 2

Supplementary Material 3

## Data Availability

The datasets used and analysed are available from the corresponding author on reasonable request.

## Abbreviations

ABF: Any breastfeeding
ABF-Q: Any breastfeeding questionnaire
EBF: Exclusive breastfeeding
EBF-Q: Exclusive breastfeeding questionnaire
pp: Postpartum
RQ: Recall questionnaire
WHO: World Health Organisation
