# Supplementary material for: Maternal recall of exclusive and any breastfeeding duration during the first 6 months- an examination of retrospective accuracy at 12 months within a large prospective breastfeeding survey in Germany
Source: Int Breastfeed J. 2026 Jan 8;21:10. doi: 10.1186/s13006-025-00808-3 (PMC12849334; doi:10.1186/s13006-025-00808-3)
Supplement: Supplementary file 1 — Supplementary Material 1 [file 13006_2025_808_MOESM1_ESM.pdf]

Additional Material 1

Scenarios for timing of questions on breastfeeding duration within the basic SUSE-II-study design

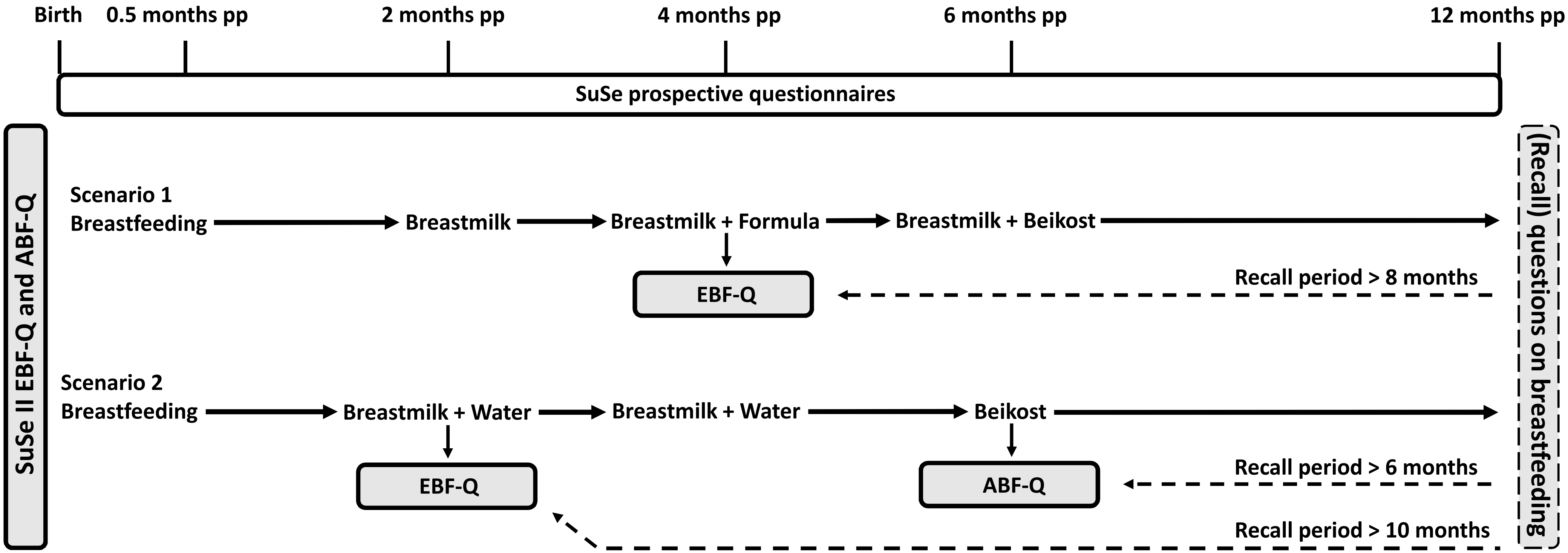

Legend

Scenario 1: When a mother stated only breastmilk as the actual nutrition and no other fluids or foods in the regular questionnaire at 2 months, she did not receive an EBF-Q or ABF-Q. If however, breastmilk + formula were reported at 4 months, she did receive the EBF-Q immediately. If she continued partial breastfeeding beyond 6 months, she never received the ABF-Q.

Scenario 2: When a mother reported breastmilk and water (or other fluids) in the regular 2-month questionnaire, she did receive the EBF-Q. If at 6 months, she no longer reported breastmilk but formula (and eventually complementary food/Beikost), she received the ABF-Q.

pp: post partum; EBF-Q: exclusive breastfeeding questionnaire (reference duration); ABF-Q: any breastfeeding questionnaire (reference duration); Beikost: complementary feeding
