# Supplementary figures and images for: Maternal recall of exclusive and any breastfeeding duration during the first 6 months- an examination of retrospective accuracy at 12 months within a large prospective breastfeeding survey in Germany
Source: Int Breastfeed J. 2026 Jan 8;21:10. doi: 10.1186/s13006-025-00808-3 (PMC12849334; doi:10.1186/s13006-025-00808-3)

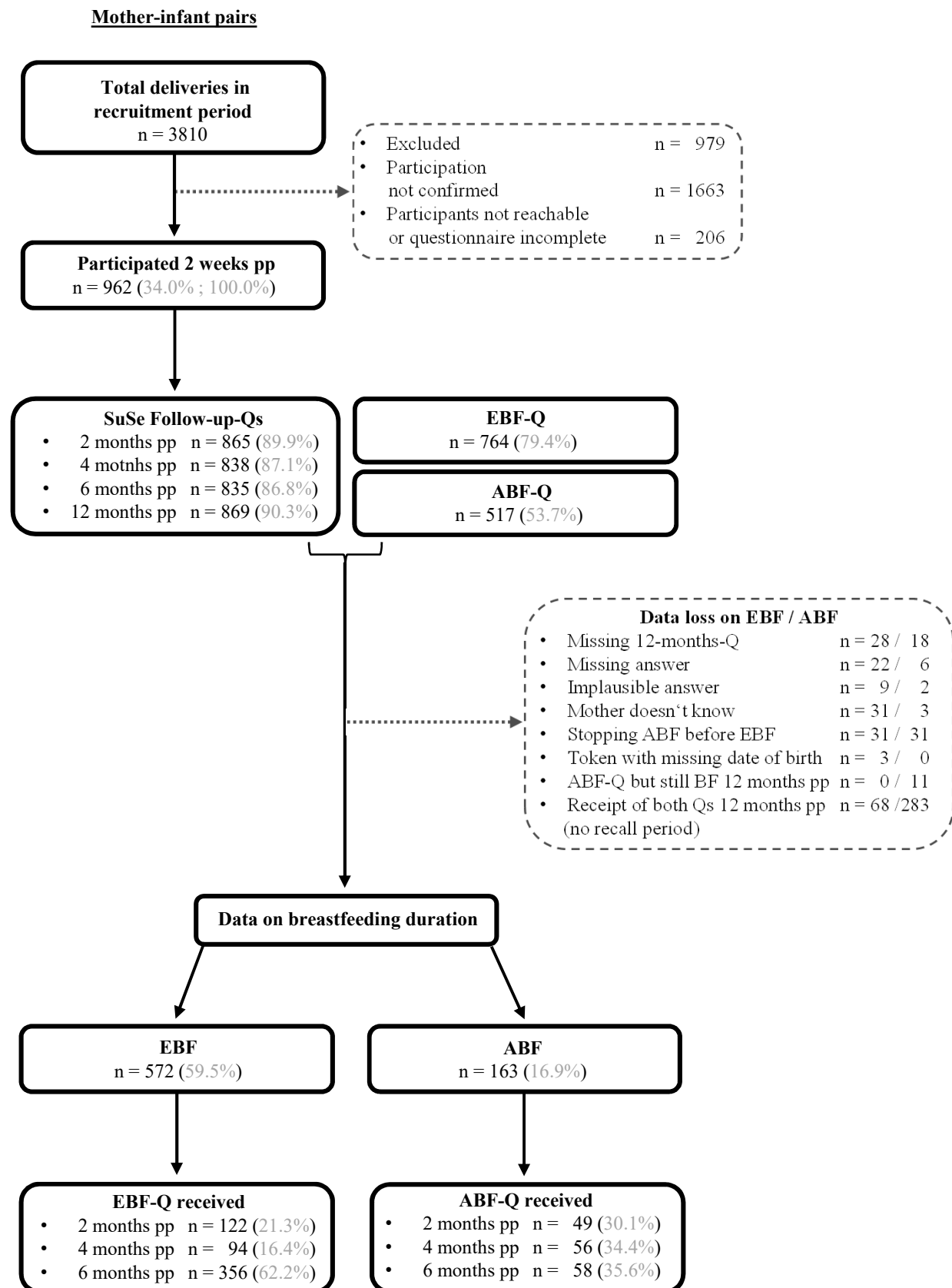

Supplement: Supplementary file 2 — Supplementary Material 2 [file 13006_2025_808_MOESM2_ESM.pdf]
