# Supplementary material for: Maternal recall of exclusive and any breastfeeding duration during the first 6 months- an examination of retrospective accuracy at 12 months within a large prospective breastfeeding survey in Germany
Source: Int Breastfeed J. 2026 Jan 8;21:10. doi: 10.1186/s13006-025-00808-3 (PMC12849334; doi:10.1186/s13006-025-00808-3)
Supplement: Supplementary file 3 — Supplementary Material 3 [file 13006_2025_808_MOESM3_ESM.docx]

**Additional file 1** (Figure, pdf)

Title: Design of timing of questions on breastfeeding duration within the basic SuSe-II-study design

Legend: pp: postpartum; EBF-Q, exclusive breastfeeding questionnaire (reference duration); ABF-Q, any breastfeeding questionnaire (reference duration); Beikost: complementary feeding

**Additional file 2** (Figure, pdf)
Title: Flowchart of recruitment of the analysed sample within the SuSe-II study

Legend: ABF, any breastfeeding; ABF-Q, any breastfeeding questionnaire; BF, breastfeeding; EBF, exclusive breastfeeding; EBF-Q, exclusive breastfeeding questionnaire; Qs, questionnaires
